# *Enterococcus* spp. in fish: Analysis of the presence and resistance in samples from Tri-City, Poland

**DOI:** 10.1371/journal.pone.0306826

**Published:** 2024-07-09

**Authors:** Anita Kukułowicz, Izabela Steinka, Maria Gardocka

**Affiliations:** Faculty of Management and Quality Sciences, Department of Quality Management, Gdynia Maritime University, Gdynia, Poland; Universidad Autonoma de Chihuahua, MEXICO

## Abstract

The growing concern over antibiotic resistance in foodborne pathogens necessitates comprehensive assessments of its prevalence and associated risks in various food products. The present study aimed to assess the occurrence of *Enterococcus* spp. in samples of fish purchased at various points of sale in the Tricity region. The selection of products (n = 74) was based on their availability and included both fish caught in the Baltic region and products imported from, Vietnam, China, Norway, and European Union (EU) countries. For bacterial isolation, samples were inoculated into selective broth, and the growth of enterococci was assessed based on turbidity. Positive cultures were confirmed by a change in color in bromocresol purple broth and were isolated on Slanetz-Bartley agar. Bacteria were present in all tested samples regardless of the degree of raw material processing as follows: frozen (F)– 55%, fresh/raw (FS)– 70.6%, thawed (DF)– 30%, smoked (S)– 50%, and the packaging methods, modified atmosphere packaging (MAP)– 34.4%, unit packaging (UP)– 75%, and sold in bulk (SB)– 76.9%, with an overall frequency of occurrence of 58.1%. The number of bacteria ranged from not detected to 4.28-log cfu/g, with the lowest mean values for thawed fish and those packed in MAP. Tests conducted on 24 strains isolated from samples showed their varied sensitivity to tetracyclines. Single cases of multidrug resistance of the tested strains were also observed. The conducted statistical analysis did not show statistically significant differences in the count of enterococci based on the origin, degree of processing, or packaging (p < 0.05). Moreover, differences in strain sensitivity to ampicillin were observed. Detected cases of resistance, especially to tetracycline, require careful monitoring and action to limit the health risks associated with resistant bacterial strains in food products.

## Introduction

Enterococci are often found in vegetables, plant matter, and various food products, especially those of animal origin. Due to environmental contamination, including surfaces, they can thrive in raw food and multiply during fermentation, and may also contaminate processed food products [1–3]. Enterococci can contribute to food spoilage by producing thermostable amines such as tyramine, histamine, phenylethylalanine, cadaverine, and putrescine. These compounds have the potential to trigger allergic reactions or even food poisoning [4]. In recent years, enterococci have become significant pathogens in hospital settings, particularly affecting individuals with compromised immune systems. Regarded as opportunistic pathogens, they are the second most common cause of hospital-acquired infections, especially urinary tract infections and wounds [3,5]. The two most common human pathogens of the *Enterococcus* genus are *E*. *faecalis* and *E*. *faecium* [2]. The increase in the number of antibiotic-resistant strains of enterococci poses a serious challenge to both medical practice and public health [5]. It is estimated that 30% of enterococcal infections in hospitals are caused by vancomycin-resistant strains [3]. These bacteria can survive mild pasteurization at a temperature of 62.5°C for 20 minutes [6]. *E*. *faecalis* inhabits the intestines of various animal species and is one of the most commonly encountered species in the human intestine. Together with *E*. *faecium*, they constitute up to 1.0% of the microbial mass colonizing the human gastrointestinal tract [7,8]. In humans, they also colonize the urinary tract and oral cavity. They can be isolated from various sources such as food, plants, water, and soil due to fecal contamination [2].

### Characteristics of enterococci

Enterococci demonstrate a positive reaction in Gram-staining tests, appearing in microscopic preparations singly, in pairs, or in short chains. Unlike some other Gram-positive bacteria, they typically do not possess flagella or form standard capsules. Additionally, they do not exhibit catalase activity. They are characterized by a significant tolerance to various environmental conditions. They can grow at temperatures ranging from 10 to 45°C, at pH levels from 4.5 to 9.6, and in the presence of 6.5% NaCl. Enterococci demonstrate the ability to hydrolyze esculin and tolerate high concentrations of bile, even up to 40% [5,9,10].

### Antibiotic-resistant enterococci in food

The presence of enterococci in food is the result of contamination caused by low hygiene levels [6,11]. Fish and seafood can be habitats for enterococci originating from rivers, lakes, and seawater, which are often contaminated with municipal sewage and aquaculture effluents [12]. Enterococci have also been isolated from crustaceans (shrimp), fish intestines, fish (salmon and turbot), and processed fish products. *E*.*faecium*, *E*. *faecalis*, *E*. *durans*, *E*. *raffinosus*, *E*. *avium*, and *E*. *malodoratus* have been isolated from both aquatic habitats and traditional or industrially processed fishery products [13]. Consuming food carrying virulence potential constitutes a significant source of infections [14]. *Enterococcus* spp., which are especially present in livestock and foods of animal origin, exhibit resistance to antibiotics [2]; however, few strains of *E*. *faecium* resistant to antibiotics originate precisely from livestock [15]. *E*. *faecium* is capable of growth even at very low temperatures between 0.1°C and 53.4°C; therefore, refrigerated food storage does not provide sufficient protection against bacterial growth [6]. The presence of *E*. *faecium* in food is undesirable mainly due to the easy development of bacterial resistance to antibiotics (vancomycin and tetracycline) [6]. The use of antibiotic growth promoters in livestock leads to antibiotics or their transformation products entering the organs and tissues of animals, and consequently into food products of animal origin. Isolates of *E*. *faecalis* and *E*. *faecium* obtained from foods of animal origin show resistance to antibiotics used in hospital and non-hospital treatment, including tetracyclines, linezolid (fish, fish products), and norfloxacin (cheese, deli meats) [16] Antibacterial agents are used in many sectors of agriculture, including aquaculture, to treat diseases of aquatic animals or to prevent infection [17,18].

### Antibiotic resistance

*Enterococcus* spp. possess a significant ability to transfer and acquire resistance genes, produce biofilms and virulence factors, promote inflammatory processes, and develop resistance to antimicrobial drugs [19–21]. *Enterococcus* spp. demonstrate resistance to most cephalosporins and all semi-synthetic penicillins, and they are resistant to low concentrations of penicillin and ampicillin [22]. Additionally, enterococci naturally exhibit resistance (*in vivo*) to clindamycin, trimethoprim-sulfamethoxazole, and low concentrations of aminoglycosides [23]. Enterococci have the capability to acquire and accumulate resistance to chemotherapeutics and antibiotics through the exchange of genetic material, which is found in plasmids or transposons [24].

Data on the presence of antibiotic-resistant enterococci in fish that is available for sale in the markets of the Tricity area of Poland are scarce. The aim of this study was to attempt to evaluate the presence of antibiotic-resistant enterococci in fish obtained from points of sale located in the Tricity area of Poland.

## Materials and methods

### Study area

Tri-City, is a metropolitan area in northern Poland consisting of three cities: Gdansk, Gdynia, and Sopot (188340E, 548270N). They are situated adjacent to one another in a row on the coast of Gdansk Bay in the Baltic Sea. The western part of Gdansk Bay is formed by the shallow waters of the Bay of Puck. The southeastern part is the Vistula Lagoon, separated by the Vistula Spit and is connected to the open sea by the Strait of Baltiysk. The choice of Tri-City as the research area stemmed from its established position as one of the most desirable tourist destinations in Poland, as well as its association with fisheries and the longstanding tradition of consuming aquatic products among the local community.

### Material

**Fig 1:**
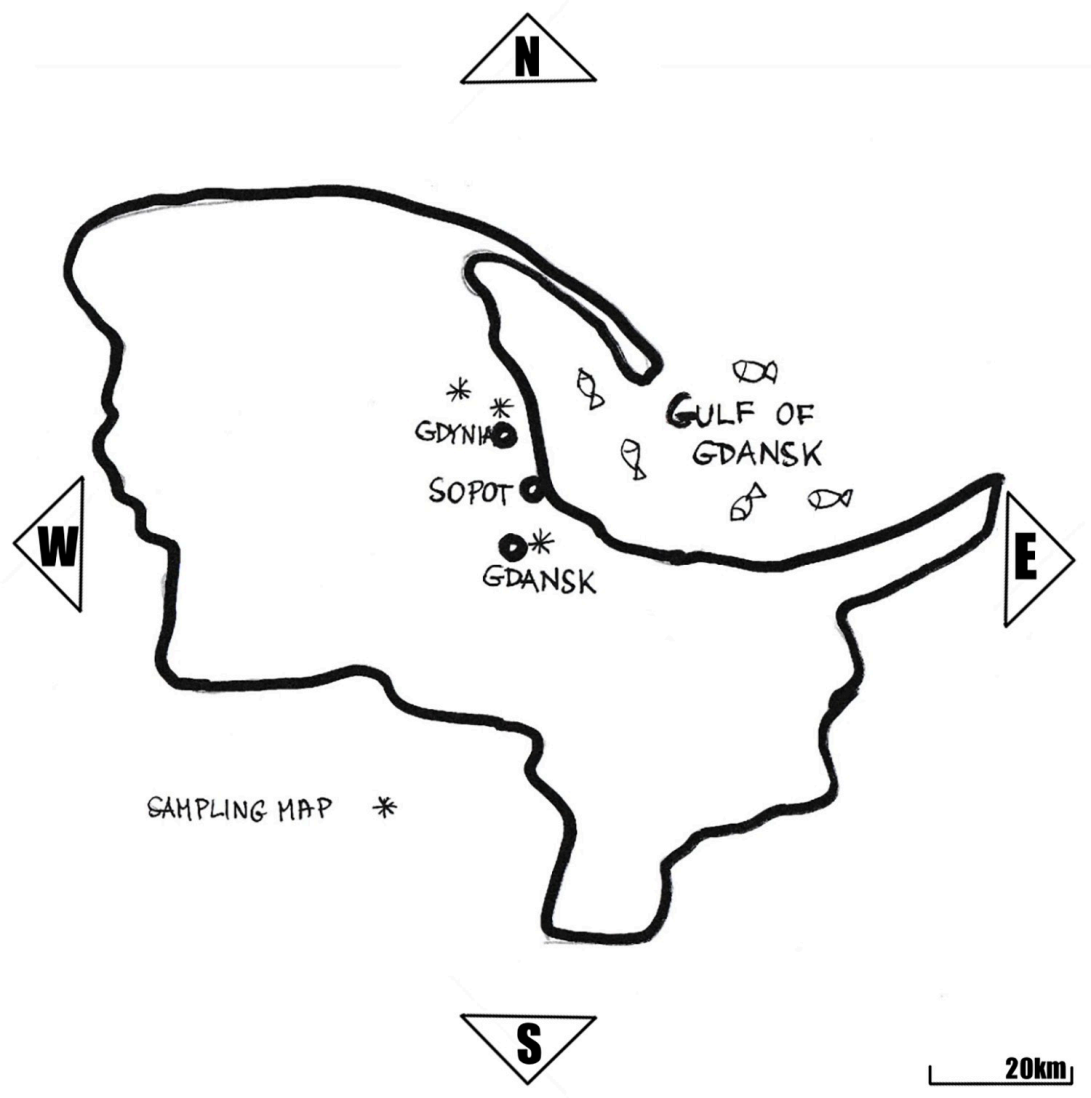
Sampling map.

Tests were conducted on fish purchased from various points of sale. The analyzed products (n = 74, Table 1) were acquired based on their availability in the Tri-City region (Fig 1). Some of the products originated from domestic catches in the Baltic Sea Region, while the remaining aquatic products were imported from countries such as Vietnam, China, Norway, and the EU.

**Table 1:** Types of tested products.

**Place of origin**	
Asian Countries (Vietnam + China) (AC)	n = 15
European Countries (UE + Norway) (EC)	n = 26
Poland (PL)	n = 33
**Degree of processing**	
Frozen (F)	n = 20
Frash/Raw (FS)	n = 34
Thawed/ Defrosted (DF)	n = 10
Smoked (S)	n = 10
**Packaging type**	
Modified Atmosphere Packaging (MAP)	n = 32
Individually/unit packaged (UP)	n = 16
Sold in bulk (SB)	n = 26

Number of samales (n).

The products were displayed on open refrigerated counters (without covers) and in self-service refrigerators. Purchased items were transported to the microbiological laboratory in a thermal insulation bag to maintain the continuity of the so-called ‘cold chain of distribution.’ The transportation of samples typically took about 45 minutes. Samples were analyzed immediately upon reaching the lab orator.

### Isolation of enterococci

For isolation, samples were inoculated onto a selective broth medium with azide and dextrose (Merck). The medium was incubated for 24–48 hours at 35°C. The turbidity of the broth was assessed as an indicator of enterococci growth. Positive broth cultures were then inoculated into bromocresol purple azide broth (Merck) and incubated at 36+/-1°C for up to 48 hours. The presence of turbidity and the color change from purple to yellow in the bromocresol purple azide broth tube confirmed the presence of enterococci (Merck). For further isolation of enterococci, dilutions of the bacterial suspension were plated on Slanetz-Bartley agar (Merck). After 48 hours of incubation at 37°C, typical dark red or maroon colonies were preliminarily identified as enterococci. For identification purposes, Gram staining, catalase tests on glass slides, gas production from mannitol, and growth in the presence of 6.5% NaCl were employed Goloś-Prądzyńska et al. [4], Soares et al. [2], and Chajęcka-Wierzchowska et al. [25].

### Antimicrobial-susceptibility testing

For all obtained microbiological isolates (n = 24), antimicrobial susceptibility was carried out using four antimicrobial agents (BioMaxima S.A, Gdansk, Poland) including ampicillin (10 μg), erythromycin (15 μg), tetracycline (30 μg), and vancomycin (30 μg). The Kirby–Bauer disk-diffusion method was used for testing, according to the Clinical and Laboratory Standards Institute (CLSI) [26]. Each *Enterococcus* isolate was inoculated onto plates with Mueller–Hinton II agar (BioMaxima S.A, Gdansk, Poland) impregnated with various antibiotic disks in different concentrations as described above at the 0.5 McFarland standard. The plates were then incubated at 37°C for 18–24 h, and the zone of inhibition formed around each disk was measured according to the CLSI guidelines and the samples were registered as sensitive or resistant, including the intermediate.

### Statistical analysis

Only positive results were used for the statistical analysis. Two outlier values, identified using the Grubbs test, were removed. The relationship between the presence of *Enterococcus* spp. in samples and their origin, processing degree, and packaging method was determined using parametric ANOVA (Analysis of Variance) for main effects. To assess the influence of the relationship between qualitative variables and the presence of antibiotic-resistant *Enterococcus* spp. in samples, a chi-square test was applied. The significance level was set at p < 0.05. Data analysis was performed using Statistica software (StatSoft, Inc.) [27].

## Results

### Total microbial level of *Enterococcus* spp.

The frequency of *Enterococcus* spp. occurrence in the examined fish samples, categorized by place of origin, degree of processing, and packaging type, is presented in Table 2. The count of *Enterococcus* spp. in the examined fish and seafood samples ranged from not detected to 4.27-log cfu/g. The lowest average values were obtained for DF, followed by those originating from EC and packaged using the MAP system, at 1.36±0.62, 1.94±0.62 and 1.90±0.83 log cfu/g, respectively. The highest level (4.27 log cfu/g) of contamination with *Enterococcus* bacteria was found in individually packaged frozen salmon from Poland, followed by bulk-sold fresh shrimp (3.84 log cfu/g) and individually packaged frozen hake (3.62 log cfu/g) from the AC.

**Table 2:** Descriptive statistic (mean log cfu /g ±SD) on positive samples.

Origin	Number of positive/negative samples	mean±SD [log cfu/g]
AC	9(15)– 60.0%	2.26±1.10
EC	16(26)– 61.5%	1.94±0.74
PL	18(33)– 54.5%	2.18±0.87
total	43(74)– 58.1%	2.11±0.87
Degree of processing		
F	11(20)– 55.0%	2.32±1.01
FS	24 (34)– 70.6%	2.14±0.87
DF	3(10)– 30.0%	1.36±0.62
S	5(10)– 50.0%	1.98±0.49
Packaging type		
MAP	11(32)– 34.4%	1.90±0.83
SB	20(26)– 76.9%	2.28±0.92
UP	12(16)– 75.0%	2.12±0.87

The conducted statistical analysis did not show statistically significant differences in the count of enterococci based on the origin (F = 0.2027; p = 0.8175), degree of processing (F = 0.5110; p = 0.6773), or packaging method (F = 0.1457; p = 0.8649). Among the examined fish samples, approximately 9.5% exhibited enterococci counts ranging from 3.23 to 4.27-log cfu/g. Approximately 42% of the samples showed no presence of these bacteria.

### Antimicrobial-susceptibility characterization

**Fig 2:**
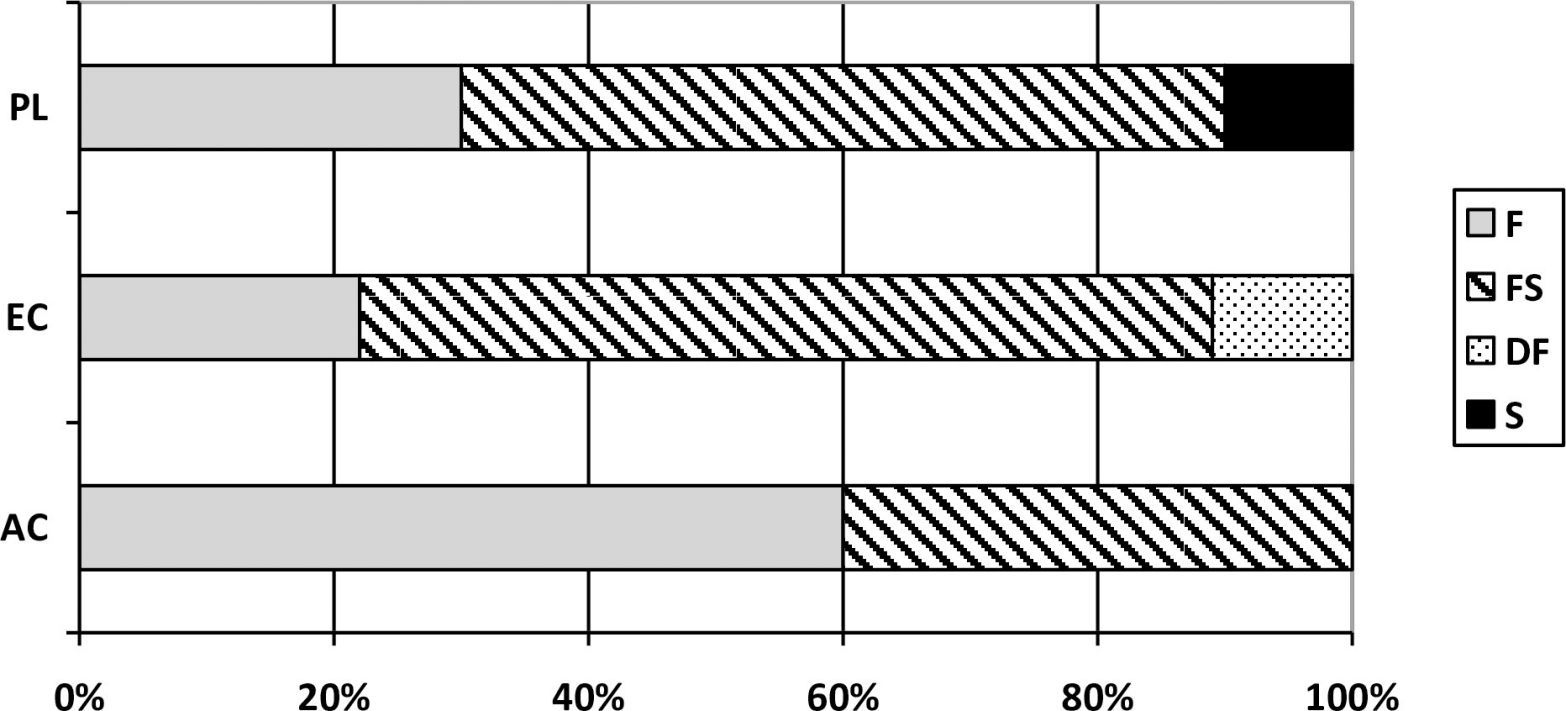
Analysis of antibiotic resistance assessment percentage by place of origin and processing degree.

Out of the 74 analyzed samples, 32.4% (n = 24) exhibited growth of *Enterococcus* spp., allowing for the preparation of an appropriate suspension (density of 0.5 on the McFarland scale), and antibiotic resistance was assessed for these isolated strains (Fig 2).

Based on the obtained values of growth-inhibition zones around the antibiotic disk, the tested strains were classified as resistant or susceptible. Table 3 presents a summary of the number of strains showing susceptibility and resistance to the tested antibiotics. Among the analyzed *Enterococcus* spp. strains, 15 exhibited resistance to tetracycline, while only 2 showed resistance to erythromycin.

**Table 3:** Summary of antibiotic susceptibility of *Enterococcus* spp. strains isolated from fish samples (n = 24).

ANTIBIOTIC (antibiotic content in disk)	NUMBER OF STRAINS SHOWING:		
	sensitive	intermediate	resistant
ampicillin (10μg)	14	0	10
erythromycin (15μg)	16	6	2
tetracycline (30μg)	4	5	15
vancomycin (30μg)	17	2	5

One isolated strain from fresh perch (PL) proved to be resistant to all four antibiotics, one from fresh herring (EC) was resistant to three (ampicillin, erythromycin, and tetracycline), while five strains showed resistance to ampicillin and tetracycline, and four to tetracycline and vancomycin. Three strains isolated from FS exhibited susceptibility to all tested antibiotics. After comparing all samples tested based on their place of origin, degree of processing, and type of packaging, it was found that ampicillin-sensitive *Enterococcus* spp. strains were more prevalent in FS samples (χ2 = 10.7755; p = 0.0130). The remaining dependencies were found to be not statistically significant (p > 0.05).

## Discussion

Bacteria belonging to the genus *Enterococcus* spp. constitute one of the most abundant groups of microorganisms in raw and animal-derived food products. This is associated with their high adaptability to the changing environment during food production and storage [28]. The frequency of occurrence of enterococci in the analyzed fish samples was 58.1% (43/74). Igbinosa and Beshiru [10] reported a lower occurrence rate (8.19%) of these bacteria in ready-to-eat shrimp samples, meanwhile, Külahci and Gündoğan [29] found that over 21% of fresh fish were contaminated with enterococci. In this study, it was demonstrated that over 70% of the analyzed FS were contaminated with these bacteria at an average level of 1.48-log cfu/g (3.0×10^1^ cfu/g). Mendoza, Aquino, and Reyes [30] found enterococci in their tilapia samples at an average level of 1.89 x10^7^ cfu/g, while Çardak et al. [13] observed the presence of enterococci ranging from 1.0 to 2.5-log cfu/g in less than 10% of the analyzed raw and processed seafood samples. Higher contamination of raw materials with enterococci may result from contamination during evisceration due to poor personal hygiene, poor processing hygiene, or poor water quality [13,31]. Processing technologies (freezing and smoking) can reduce the bacterial load of food [32], as observed in this study (Table 2). Differences in the number of enterococci contaminating the tested products depending on the packaging system (Table 2) may result from cross-contamination during processing or preparation, during which bacteria can be transferred from raw fish, contaminated surfaces, or equipment [13]. Enterococci can cause food spoilage. By producing thermostable amines such as tyramine, histamine, phenylethylalanine, cadaverine, and putrescine, they can cause allergic reactions or intoxications [4].

Many factors can influence the pathogenicity of *Enterococcus* spp., such as their ability to colonize the gastrointestinal tract or their adhesion to various extracellular matrix proteins or epithelial cells. Pathogenic *Enterococcus* spp. (*E*. *faecalis* and *E*. *faecium*) are widely distributed in farmed tilapia and are a cause of sepsis, urinary tract infections, and wounds in humans [33]. According to Regulation (EC) No 852/2004 [34], food business operators are required to comply with microbiological criteria. Currently applicable regulations in Poland do not specify permissible limits for enterococci in fish and their environment. In the current Commission Regulation (EC) No 2073/2005 [35], limits apply only to *Salmonella*, *E*. *coli*, histamine, and coagulase-positive staphylococci.

Antibiotic-resistant enterococci are present in raw food of animal and plant origin [36]. In the studies by Igbinosa and Beshiru [10], the resistance profile of isolated *Enterococcus* spp. to penicillin, erythromycin, tetracycline, and vancomycin was 88.1%, 49.2%, 45.8%, and 37.3%, respectively. In our own research, the overall resistance rate was highest for tetracycline (62.5%), while the lowest resistance rate was observed for erythromycin (8.3%). Çardak et al. [13], in addition to a high percentage (> 50%) of resistance to tetracycline, streptomycin, gentamycin, and chloramphenicol, also showed a high percentage of resistance to erythromycin in enterococcal strains isolated from raw and processed seafood samples. Isolates of *E*. *faecium* and *E*. *faecalis* from animal-derived food products exhibit resistance to antibiotics used in both outpatient and hospital treatment, including ciprofloxacin, norfloxacin, and tetracycline [36], and resistance to tetracycline is one of the most commonly reported among enterococci isolated from food [37]. Similar to Tansuphasiri, Khaminthakul, and Pandii [38], the majority of isolates (75%) in samples from frozen fish were resistant to tetracycline in this study. In our research, it was shown that the resistance rate for vancomycin was approximately 21%. Vancomycin-resistant enterococci may exhibit pathogenic properties and cause bacteremia or endocarditis in humans, although it is uncertain whether animals are their source and to what extent this occurs [15].

In our studies, multidrug resistance to at least three different classes of antibiotics was detected in two (14.3%) strains isolated from FS. This result was significantly lower than that obtained in the study by Ben Said et al. [12]. Tansuphasiri, Khaminthakul, and Pandii [38] showed that the majority of isolates from frozen samples were multidrug-resistant (50%). Araujo et al. [5], based on research on the resistance and virulence profile of enterococci isolated from ponds and reservoirs in southern Brazil, identified a total of 79 enterococcal strains, with *E*. *faecalis* being the most frequently isolated species (44.3%). Sixty-five strains (82.3%) were resistant to at least one of the antimicrobial agents tested, while 27 strains (34.2%) exhibited multidrug resistance. The overall resistance rate of the bacteria they isolated was 36.7% for erythromycin and 30.4% for tetracycline [5]. Boss et al. [39] found that the highest resistance rates were related to *E*. *faecalis* to tetracycline (16%) in their study of samples from salmon, pangasius, shrimp, and oysters. Our research showed that 62% of the isolated strains were resistant to this antibiotic.

There is a risk that bacteria exhibiting resistance that originate from wastewater or water used in aquaculture can enter the sea and transfer antibiotic-resistance genes to the bacterial microflora present in local fish populations [12]. Due to their highly adaptive abilities, enterococci present in food are capable of transient or permanent colonization of the gastrointestinal tract, increasing the risk of gene transfer to the gut microbiota. Most often, these strains show resistance to streptomycin, erythromycin, tetracycline, and rifampicin [11]. Literature data report that many strains isolated from food and food products (pasteurized milk, soft cheese, raw meat, and vegetables) have similar virulence determinants in their genomes as those found in clinically derived strains [20]. The same virulence genes can make strains isolated from food a risk of infection for consumers, especially for those with weakened immunity.

One of the limitations of this study was the small sample size in some of the groups, as well as the lack of species-level characterization of *Enterococcus*. The research will be continued, and the sample size will be increased. Additionally, the analyses will be expanded to include species-level characterization of *Enterococcus*.

## Conclusion

These studies demonstrate how quickly bacteria develop resistance to drugs, becoming a common and serious threat to human health. They also highlight the fact that aquatic products may serve as a reservoir for pathogenic bacteria of the genus *Enterococcus* spp. carrying antibiotic-resistance traits. Statistical analyses revealed significant variability in antibiotic sensitivity, particularly regarding ampicillin. Detected cases of resistance, especially to tetracycline, require careful monitoring and action to limit the health risks associated with resistant bacterial strains in food products. The presence of multidrug-resistant strains in the tested fish is also concerning, especially as they can become a source of resistance genes for other bacteria.

## Supporting information

S1 Dataset Minimal data set. This file contains a data set encompassing the results of microbiological studies and the antibiotic resistance profiles of the isolated strains to selected antibiotics.
(XLSX)
